# Случай синдрома резистентности к тиреоидным гормонам вследствие ранее не описанной мутации в гене <i>THRА</i>

**DOI:** 10.14341/probl13541

**Published:** 2025-07-22

**Authors:** Ю. Л. Скородок, Т. С. Грабчак, Е. В. Плотникова, Е. Н. Суспицын, И. Ю. Иоффе, А. В. Кожевникова, В. Д. Забинский, Д. О. Иванов

**Affiliations:** Санкт-Петербургский государственный педиатрический медицинский университет; Санкт-Петербургский государственный педиатрический медицинский университет; Санкт-Петербургский государственный педиатрический медицинский университет; Санкт-Петербургский государственный педиатрический медицинский университет; Санкт-Петербургский государственный педиатрический медицинский университет; Санкт-Петербургский государственный педиатрический медицинский университет; Санкт-Петербургский государственный педиатрический медицинский университет; Санкт-Петербургский государственный педиатрический медицинский университет

**Keywords:** гипотиреоз, резистентность, тиреоидные гормоны, THRA, мутация

## Abstract

Синдром резистентности к тиреоидным гормонам (СРТГ) характеризуется пониженной чувствительностью периферических тканей к активным формам тиреоидных гормонов. В данной статье описан клинический случай пациента с симптомами гипотиреоза, но с низконормальным уровнем свТ4 при нормальных ТТГ и общего Т3. Использование метода массового параллельного секвенирования позволило выявить ранее не описанный гетерозиготный вариант в гене THRA c.1198C>G (p.Leu400Val). Сопоставление результатов молекулярно-генетического исследования и фенотипа пациента позволило верифицировать диагноз: «СРТГ типа α». Данный клинический случай является первым описанием этой патологии в России. Заместительная терапия левотироксином не сопровождалась значимым улучшением клинической картины, а использование супрафизиологических доз привело к улучшению липидограммы, однако сопровождалось появлением некоторых симптомов тиреотоксикоза.

## Abstract (en)

Thyroid hormone resistance (THR) syndrome is characterized by decreased sensitivity of peripheral tissues to active forms of thyroid hormones. We present a clinical case of a patient, demonstrating symptoms of hypothyroidism while having subnormal FT4 and normal TSH and total T3 levels. Massive parallel sequencing allowed us to identify a previously undescribed heterozygous THRA c.1198C>G (p.Leu400Val) variant that arose de novo. The results of genetic testing corresponded well with the patient’s phenotype that made it possible to verify the diagnosis of THR. Levothyroxine replacement therapy did not resulted in a significant clinical improvement; the use of supraphysiological doses led to refinement of the lipid profile but was accompanied by the appearance of some symptoms of thyrotoxicosis.

## АКТУАЛЬНОСТЬ

Синдром резистентности к тиреоидным гормонам (СРТГ) — наследственное заболевание, характеризующееся пониженной чувствительностью тканей-мишеней к гормонам щитовидной железы (ГЩЖ) [1]. Действие ГЩЖ реализуется преимущественно через Т3-рецепторы, которые, в свою очередь, взаимодействуют с ядерными рецепторами тиреоидных гормонов, кодируемыми генами THRA и THRB. РНК-продукт каждого из генов подвергается альтернативному сплайсингу для создания подтипов рецепторов (TRα1, TRα2, TRβ1, TRβ2 и TRβ3) с различным распределением в тканях. TRα1 является преобладающей изоформой в мозге, костях, сердце, кишечнике и печени, а подтип TRβ1 — в головном мозге, печени и почках [2]. Экспрессия подтипа TRβ2 ограничивается в основном гипоталамусом и тиреотрофами гипофиза [2][3]. Подтип TRα2 недостаточно изучен, идентифицирован в мозге и сердце, однако его способность связывать ГЩЖ сомнительна [4]. Экспрессия подтипа TRβ3 описана только в почках, печени, селезенке и легких крыс [5].

Тяжесть клинического фенотипа пациентов с СРТГ, обусловленным дефектами THRA, связана, по-видимому, с местоположением и типом мутации [6][7][8]. Наиболее часто наблюдаемые проявления заболевания: анемия, запоры, задержка роста и психомоторного/психоречевого развития. Уровни свободного T3 (свТ3) могут быть от нормальных до высоких, свободного T4 (свТ4) и реверсивного Т3 (рT3) — от низконормальных до низких при нормальном ТТГ.

Впервые СРТГ, обусловленный de novo нонсенс-мутацией THRА, был описан в 2012 г. [8]; с тех пор идентифицировано более 30 различных мутаций в этом гене. Большинство пациентов имели задержку роста, специфические черты лица, легкую или умеренную умственную отсталость, нормохромную анемию, брадикардию, запоры, дислипидемию [8][9][10][11]. Эти симптомы отражают характер экспрессии THRА преимущественно в центральной нервной системе, желудочно-кишечном тракте, сердечной и мышечной ткани, а также в костях и печени. Мутации располагались в 8 и 9 экзонах THRA, кодирующих лиганд-связывающий домен C-конца рецептора, что приводило к нарушению связывания Т3 с рецепторами, в результате вызывая клинические симптомы гипотиреоза. Большинству пациентов была рекомендована терапия левотироксином в стандартных дозах, которая не привела к улучшению. У отдельных пациентов на фоне терапии высокими дозами левотироксина удавалось добиться нормализации спектра липидов, снижения тяжести запоров, ускорения линейного роста, увеличения расхода энергии в покое для ограничения набора веса и улучшения самочувствия [12]. Однако этот терапевтический подход потенциально может быть связан с тиреотоксическими побочными эффектами, в том числе со стороны сердечно-сосудистой системы [13]. В нескольких экспериментальных разработках специфической терапии СРТГ (мутации в THRA) была показана эффективность агониста TRα1 (СО24) и гистонацетилаз, однако дальнейшие исследования на людях не проводились [14][15].

Предлагаемый клинический случай является первым описанием данной патологии в России.

## ОПИСАНИЕ СЛУЧАЯ

Пациент в возрасте 1 года 1 месяца обратился с жалобами на задержку психомоторного развития, отечность лица, запоры.

Из анамнеза жизни известно, что ребенок от 2-й беременности путем ЭКО (1-я беременность замершая, у плода трисомия по 16-й хромосоме) на фоне анемии легкой степени, маловодия, миопии слабой степени. Роды первые, на 41-й неделе, со стимуляцией. При рождении масса — 3610 г (SDS +0,53), длина —54 см (SDS +2,17), 9/10 баллов по шкале Апгар. Успешно прошел неонатальный скрининг (ТТГ — 2,64 мМЕ/л), находился на грудном вскармливании до 1 года.

Наследственность: рост матери — 175 см, отца — 185 см. У бабушки по линии матери гиперхолестеринемия; у бабушки по отцовской линии и прабабушки по материнской линии острые нарушения мозгового кровообращения и острый коронарный синдром.

С рождения у ребенка темповая задержка психомоторного развития, синдром диффузной мышечной гипотонии, в связи с чем наблюдается неврологом. Состоит на учете у офтальмолога: стеноз носослезного канала слева, осложненный дакриоциститом. Отмечается склонность к запорам (стул 1 раз в 2–3 дня). В 10 месяцев выявлены анемия легкой степени (Hb 104 г/л; норма 105–135), повышение АСТ (90 ЕД/л; норма 15–60), КФК (390 ЕД/л; норма <228), холестерина (7,37 ммоль/л; норма <5,2). При УЗИ органов брюшной полости — умеренная гепатомегалия. В 1 год ТТГ — 2,39 мМЕ/л (норма 0,6–10,0), свТ4 — 0,73 нг/дл (норма 0,7–1,48). При обследовании в гастроэнтерологическом отделении в 1 год 1 мес лабораторные изменения сохраняются, дополнительно обнаружены повышение КФК-МВ (48 ЕД/л; норма 0–25) и ЛПНП (4,32 ммоль/л; норма <4). Отмечается брадикардия 110 уд./мин. (норма 120–125). ТТГ — 3,1 мМЕ/л (норма 0,6–10,0), свТ4 — 10,0 пмоль/л (норма 10,0–23,0), общий Т3 — 1,3 нмоль/л (норма 0,6–3,9). При УЗИ щитовидная железа в типичном месте, зоба нет (общий объем — 1,05 см³). Параллельно пациент консультирован генетиком, в ходе биохимического, электрофоретического и молекулярно-генетического исследований исключены болезни накопления (болезнь Помпе, Краббе, Фабри, Гоше, Нимана-Пика, мукополисахаридоз 1 типа), органические ацидурии, аминоацидопатии, дефект лизосомальной кислой липазы, моногенная дислипидемия. Между тем методом электрофореза выявлена дислипидемия типа 2а. Генез гиперхолестеринемии, гиперферментемии оставался неясным, пациент направлен к эндокринологу.

Осмотр эндокринолога в 1 год 1 мес (рис. 1): в контакт вступает неохотно, эмоциональный тонус снижен. Длина тела — 80 см (SDS +0,71), масса — 13,2 кг, ИМТ — 20,6 кг/м² (SDS +2,54). Отмечаются большая голова, плоская широкая переносица, гипертелоризм, выраженный поясничный лордоз, относительное укорочение конечностей, «утиная» походка. Лицо отечное, рот приоткрыт, макроглоссия. Большой родничок 2,5–3×2,5 см. Кожные покровы иктеричные. Мышечная гипотония. Щитовидная железа не увеличена, однородная. Тоны сердца ясные, ритмичные, ЧСС 108 уд./мин. Наружные гениталии сформированы правильно по мужскому типу, половое развитие I степени по TanneR. На основании клинической картины и дважды низконормального уровня свТ4 при нормальных ТТГ и общего Т3 диагностирован гипотиреоз, предположительно, центрального генеза, начата заместительная терапия левотироксином в дозе 12,5 мкг/сут.

**Figure:**
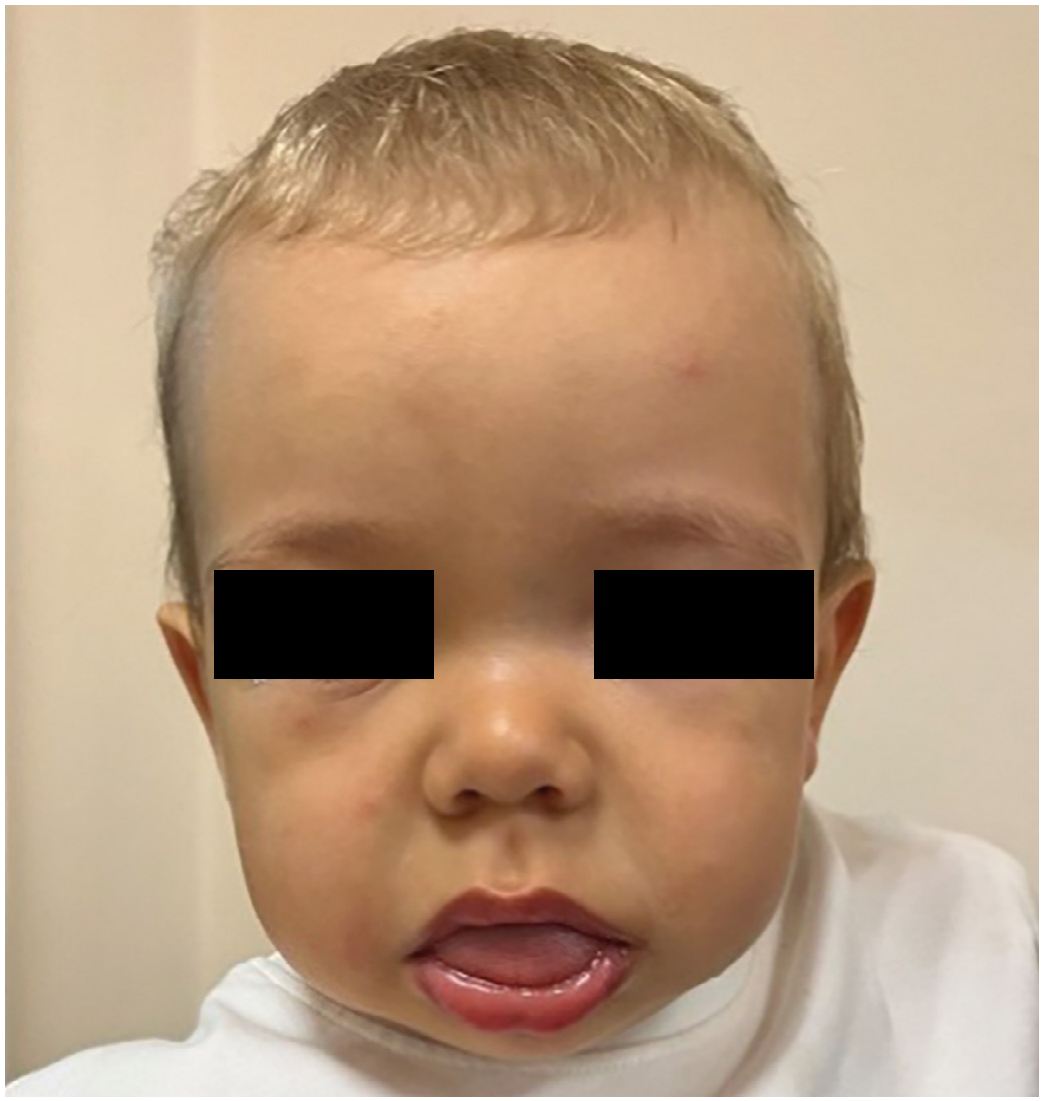
Рисунок 1. Пациент в возрасте 1 г. 1 мес.

Для уточнения причины гипотиреоза выполнено массовое параллельное секвенирование, панель «Эндом»: в 9 экзоне THRA (NM_199334.5) выявлен ранее не описанный в литературе вариант (HG38, chr17:40089421C>G, c.1198C>G) в гетерозиготном состоянии, приводящий к аминокислотной замене p.(Leu400Val). Проведена верификация мутации у родителей методом секвенирования по Сэнгеру: у матери и отца пробанда вариант не обнаружен.

При осмотре в 1 год 10 мес на фоне терапии левотироксином 37,5 мкг/сут: длина тела — 89,5 см (SDS +0,89). Скорость роста — 13 см/год (SDS +1,6). Масса тела — 16,15 кг, ИМТ — 20,2 кг/м² (SDS +2,87). Окружность головы — 50,5 см (SDS +1,86). Зубы 8/6. Ходит самостоятельно, но испытывает затруднение при вставании. Интересуется предметами, произносит отдельные слоги, понимает обращенную речь. Реакция замедленная. Голос не грубый. Сохраняется отечность лица и шеи. Тоны сердца отчетливые, ЧСС 102–106 уд./мин. Щитовидная железа в условиях затрудненного доступа не увеличена. На фоне 50 мкг/сут левотироксина (с 1 года 3 мес до 1 года 7 мес) наблюдалось незначительное смягчение клинических симптомов (запоров, отечности лица и шеи), улучшение показателей липидограммы, что сочеталось с гипертироксинемией и некоторыми проявлениями тиреотоксикоза, в связи с чем дозу препарата пришлось снизить (табл. 1). Результаты лабораторных исследований до и на фоне терапии представлены в таблице 1.

**Table:** Таблица 1. Результаты лабораторных исследований до и на фоне терапии Примечание. ТТГ — тиреотропный гормон; свТ4 — свободный тироксин; свТ3 — свободный трийодтиронин; rT3 — реверсивный трийодтиронин; Hb — гемоглобин; ЦП — цветовой показатель.

Показатель	1 год	1 год 1 месяц	1 год 2 месяца	1 год 2 месяца 21 день	1 год 3 месяца	1 год 4 месяца	1 год 7 месяцев	1 год 10 месяцев	Референсный интервал
Примечание						Жалобы: раздражителен, плохо засыпает			
ТТГ, мМЕ/л	2,39	3,1	-	0,436	0,0500	-	<0,005	<0,0083	0,7–4,17
свТ4, пмоль/л	9,4	10	10,63	10,59	11,2	16,02	16,59	13,01	10–14,29
Т3 общий, нмоль/л		1,3							0,6–3,9
свТ3, пмоль/л	-	-	-	-	-	-	8,2	6,42	4,3–6,8
rT3, пг/мл								71	83–229
Триглицериды, ммоль/л		1,02		0,83		0,67		0,72	0,34–1,13
Холестерин общий, ммоль/л	7,5	7,6		7,04		5,53		7,78	2,95–5,25
Холестерин-ЛПВП, ммоль/л		1,71		1,64		1,52		1,82	0,78–1,68
Холестерин- ЛПНП, ммоль/л		4,32		5,02		3,71		5,63	1,76–3,36
АЛТ, Ед/л	12	14			10	12	15	9	<33
АСТ, Ед/л	91	92			65		84	61	<48
Hb, г/л; ЦП		103,3; 0,82	105; 0,8		102; 0,81	110; 0,84			105–130; 0,85–1,15
Ферритин, мкг/л						11		10	12–100
Левотироксин, мкг/сут	-	-	12,5	25	37,5	50	50	37,5	

## ОБСУЖДЕНИЕ

У пациента отмечалось множество признаков гипотиреоза: задержка психомоторного развития, запоры, избыток массы тела, отечность лица и шеи, макроглоссия, гиперхолестеринемия, гепатомегалия, гипохромная анемия, особенности внешности (большая голова, широкая плоская переносица, гипертелоризм), относительное укорочение конечностей, гиперлордоз, брадикардия, снижение мышечного тонуса, мышечная слабость. Эти симптомы могут объясняться преимущественной экспрессией подтипа TRα1 в головном мозге (нарушение дифференцировки нейронов [16]), костной ткани (замедление пролиферации и дифференцировки остеобластов, хондроцитов [17]), кишечнике (укорочение ворсинок, нарушение дифференировки клеток крипт, снижение пролиферации стволовых клеток, повышение парасимпатической активности [18]), в ткани скелетных мышц (снижение скорости обмена жирных кислот, оборота митохондриального пула и потока цикла Кребса) и печени (нарушение процессов аутофагии и липофагии, β-окисления жирных кислот) [19]).

Клинические проявления, наблюдаемые у представленного пациента, совпадают с ранее описанными другими авторами [3][4][6][7][8][9][10][11]. Наиболее постоянными симптомами были особенности внешности, брадикардия и ЗПМР. Отечный синдром и избыточная масса тела отмечались у 14 из 19 пациентов, описанных в литературе. Задержка роста выявлена только у 8 пациентов, включая 3 с нонсенс- и 5 — с миссенс-мутацией. Кроме того, у одной пациентки с нонсенс-мутацией и одной обладательницы мутации, приводящей к сдвигу рамки считывания [3], наряду с признаками гипотиреоза отмечались аномалии развития костной системы (врожденный вывих бедра, дисгенезия эпифиза бедренной кости). В нашем случае (миссенс-мутация) задержка роста и патология тазобедренных суставов (УЗИ) отсутствуют.

Результаты лабораторных исследований (низконормальный уровень свТ4 при нормальных ТТГ и общего Т3) не соответствовали выраженной клинике гипотиреоза, что совпадает с описаниями подобных случаев в литературе [7][10], в части из которых в связи с подозрением на центральный гипотиреоз определение Т3 не проводили. Другие авторы указывают на высокий уровень свT3 и низкий rT3 до начала терапии [3][6][11]. У пациентов с СРТГ вследствие мутации в THRA отсутствуют изменения концентрации ТТГ в крови (поскольку TRα не экспрессируются в гипофизе) и, как следствие — повышение ГЩЖ. Между тем уровни как общего Т3, так и свT3 высоконормальные/высокие, в то время как свT4 — низконормальные/субнормальные, что приводит к увеличению соотношения T3/T4 [12]. Это, вероятно, объясняется повышенной экспрессией дейодиназы 1 типа (в исследовании на мышах с мутацией в THRA отмечалось 9-кратное повышение уровня мРНК гепатической дейодиназы 1 типа). Кроме того, активность дейодиназы 3 типа, обеспечивающей деградацию T3, у таких же экспериментальных животных была снижена до 30% от нормы [14].

Выявленная у нашего пациента дислипидемия типа 2а (повышение общего холестерина и ЛПНП) согласуется с мнением о гиперхолестеринемии как наиболее частом биохимическом изменении при СРТГ вследствие мутации в THRA [18]. У всех описанных ранее пациентов, независимо от типа мутации, отмечалась гиперхолестеринемия, что объясняется нарушениями процессов бета-окисления, митохондриального биогенеза и аутофагии в клетках печени, экспрессирующих TRα [19].

Гипохромная анемия у нашего пациента совпадает с описанными ранее случаями [3][9] и может быть объяснена снижением запасов железа (ферритина) при гипотиреозе. В нашем случае наблюдалась повышенная активность креатинкиназы, характерная для гипотиреоза, как и у всех описанных пациентов [3][6][7].

В связи с несоответствием клинической и лабораторной картины гипотиреоза низконормальному уровню свТ4 при нормальных ТТГ и общего Т3 пациенту потребовалось МГИ для уточнения генеза заболевания. Выявленный вариант THRА ранее не описан в литературе. По совокупности сведений вариант расценивается как обладающий неопределенной клинической значимостью [20], однако сопоставление фенотипа пациента с картиной THRA-ассоциированных заболеваний позволяет подтвердить СРТГ.

На фоне приема левотироксина 50 мкг/сут уровни свТ4 и свТ3 повысились до супрафизиологических, приведя к подавлению ТТГ. Согласно литературным исследованиям in vitro, сниженная транскрипционная активность мутантного рецептора может быть преодолена более высокими концентрациями свT3 [18], что проявилось у пациента значимым улучшением липидограммы. Кратковременная нормализация показателей липидного обмена может объясняться индукцией ГЩЖ аутофагии в клетках печени, экспрессирующих TRα, с последующим повышением липогенной активности печени в исследованиях in vitro [12]. Более длительную терапию левотироксином в дозе 50 мкг/сут проводить не представлялось возможным в связи с проявлениями тиреотоксикоза у пациента (нарушения засыпания, раздражительность). Однако снижение дозы сопровождалось возвратом гиперхолестеринемии, в связи с чем должна обсуждаться возможность новых попыток использования супрафизиологических доз [12] либо альтернативной фармакотерапии [9][15].

## ЗАКЛЮЧЕНИЕ

У пациентов с несоответствием выраженности симптомов гипотиреоза и отсутствия характерных изменений лабораторных показателей необходимо проведение молекулярно-генетического исследования, включающего идентификацию мутаций гена THRA. Синдром резистентности к тиреоидным гормонам, ассоциированный с ранее не описанной миссенс-мутацией THRА, привел к фенотипу гипотиреоза при низконормальном уровне свТ4 и нормальных ТТГ и общего Т3, а заместительная терапия левотироксином не сопровождалась значимым улучшением клинической картины. Подобные результаты характерны для большинства описанных пациентов с патогенными вариантами THRА. Применение супрафизиологических доз левотироксина привело к улучшению липидограммы, но сопровождалось некоторыми симптомами тиреотоксикоза, и потому является дискутабельным.

## ДОПОЛНИТЕЛЬНАЯ ИНФОРМАЦИЯ

Источники финансирования. Молекулярно-генетическое исследование выполнено при частичном содействии фонда поддержки и развития филантропии «КАФ».

Конфликт интересов. Авторы декларируют отсутствие явных и потенциальных конфликтов интересов, связанных с содержанием настоящей статьи.

Участие авторов. Все авторы одобрили финальную версию статьи перед публикацией, выразили согласие нести ответственность за все аспекты работы, подразумевающую надлежащее изучение и решение вопросов, связанных с точностью или добросовестностью любой части работы.

Согласие пациента. Пациенты добровольно подписали информированное согласие на публикацию персональной медицинской информации в обезличенной форме в журнале «Проблемы эндокринологии».

Благодарности. Выражаем благодарность Корниенко Елене Александровне, д.м.н., профессору кафедры детских болезней им. проф. И.М. Воронцова ФП и ДПО СПбГПМУ, врачу-гастроэнтерологу, за своевременный диагностический поиск и оказание врачебной помощи пациенту и Гаськовой Марине Владимировне, врачу КЛД лаборатории общей и популяционной генетики ФГБУ НМИЦ эндокринологии за квалифицированное проведение МГИ пробанду.
